# Tobacco use increases susceptibility to bacterial infection

**DOI:** 10.1186/1617-9625-4-12

**Published:** 2008-12-18

**Authors:** Juhi Bagaitkar, Donald R Demuth, David A Scott

**Affiliations:** 1 Department of Microbiology and Immunology, University of Louisville, Louisville, KY, USA; 2 Department of Pharmacology and Toxicology, University of Louisville, Louisville, KY, USA; 3 Oral Health and Systemic Disease Research Group, University of Louisville, Louisville, KY, USA

## Abstract

Active smokers and those exposed to secondhand smoke are at increased risk of bacterial infection. Tobacco smoke exposure increases susceptibility to respiratory tract infections, including tuberculosis, pneumonia and Legionnaires disease; bacterial vaginosis and sexually transmitted diseases, such as chlamydia and gonorrhoea; *Helicobacter pylori *infection; periodontitis; meningitis; otitis media; and post-surgical and nosocomial infections. Tobacco smoke compromises the anti-bacterial function of leukocytes, including neutrophils, monocytes, T cells and B cells, providing a mechanistic explanation for increased infection risk. Further epidemiological, clinical and mechanistic research into this important area is warranted.

## Review

It is well established that smokers are more susceptible than non-smokers to a plethora of chronic diseases and conditions that include stroke, vascular diseases, chronic obstructive pulmonary disease, multiple cancers, periodontal diseases, hypertension, impotence and osteoporosis. However, smokers are also significantly more susceptible to multiple bacterial infections than are non-smokers. Such infections can be life-threatening and both active smokers as well as those exposed to secondhand smoke toxins are at increased risk. This important relationship between smoking and ill-health may not be universally appreciated. Therefore, we set out to review and summarize the evidence associating tobacco smoke exposure with bacterial infections and to introduce interactions between tobacco smoke, bacteria, and the immune system that may help explain the increased susceptibility to infectious disease in smokers.

## Bacterial infections associated with tobacco smoking

### Nasopharyngeal and respiratory tract infections

Adult smokers are at increased risk of respiratory infection by several bacterial pathogens, including *Streptococcus pneumonia*, *Neisseria meningitidis*, *Haemophilus influenzae *and *Legionella pneumophila *[1-5]. Brook and Gober reported that the nasopharyngeal microflora of smokers contains fewer normal bacteria (such as α-hemolytic and nonhemolytic streptococci, and *Prevotella *and *Peptostreptococcus *species) that can interfere with colonization by selected pathogens (*S. pneumoniae*, *H. influenzae *(non-type b), *Moraxella catarrhalis*, and *Streptococcus pyogenes*) and that the nasopharyngeal microflora also contains more potential pathogens compared with those of nonsmokers[6]. Such increased carriage of potentially pathogenic species of bacteria in both adults and children was hypothesized [6] to possibly be due to enhanced bacterial binding to the epithelial cells of smokers [7] and the low number of α-hemolytic streptococci with inhibitory activity against *S. pyogenes *in the oral cavities of smokers [6,8]. More recently, the same group followed a small number of quitters (n = 20) for a period of up to 15 months post-tobacco cessation, and concluded that the high number of pathogens (*S. pneumoniae, H. influenzae, M. catarrhalis*, and *S. pyogenes*) and low number of interfering organisms found in the nasopharynx of smokers reverted to normal levels after stopping smoking [9].

When comparing patients with stable chronic obstructive pulmonary disease (COPD) with healthy controls (normal spirometry and chest radiography) Zalacain et al found current smoking to be associated (OR 3.17, 95% CI 2.50–8.00) with pulmonary infection with pathogenic bacteria – *H. influenzae*, *Streptococcus viridans*, *S. pneumoniae *and *M. catarrhalis *being the most frequently cultured species [10]. In patients with chronic obstructive pulmonary disease (COPD), bacterial exacerbations are also more prevalent among current smokers (OR 3.77, 95% CI 1.17–12.12) [11,12].

Several studies indicate that exposure to passive smoking also increases the incidence of sneezing, sore throat, cough, and frequent cough [13,14]. The incidence of group A streptococcus sore throat has been reported to be higher among children living in homes that include a tobacco smoker [15]. Thus, there is a consistent body of literature showing a significant association between tobacco smoke exposure and respiratory tract infections. More specific pulmonary diseases are now considered.

### Cystic Fibrosis

Cystic fibrosis is a life-shortening autosomal recessive disease affecting the pulmonary, gastrointestinal and genitourinary systems. Lung colonization with a succession of pathogens, such as *Staphylococcus aureus *and *H. influenzae *is likely to occur in affected individuals. Ultimately, persistent colonization by the opportunistic pathogen *Pseudomonas aeruginosa *contributes to significant morbidity and mortality in cystic fibrosis [16]. Epidemiological studies have shown that secondhand smoke exposure is associated with poor prognosis in cystic fibrosis patients [17] and that a dose-dependent relationship exists between number of cigarettes smoked and severity of disease amongst young patients [18]. In keeping with these data, mice infected with *P. aeruginosa *and exposed to tobacco smoke exhibit delayed clearance of infection and increased morbidity compared to control mice which were infected but not exposed to smoke [19].

### Pneumonia, Legionnaires' disease and bronchitis

Several studies have shown smoking to be significantly associated with the development of bronchitis and bacterial pneumonia [20]. There are dose-response relations between the current number of cigarettes smoked per day; pack-years of smoking; and time since quitting and invasive pneumococcal disease, with approximately 50% of those with invasive pneumococcal disease being cigarette smokers [2]. Recently, several authors have discussed the possibility of recommending the routine extension of pneumococcal vaccine coverage to current smokers [21,22]. There is also strong evidence that smoking is an independent risk factor for Legionnaires disease, an atypical pneumonia that usually develops 2 to 14 days after exposure to *Legionella pneumophila *[5,23]. Complications of Legionnaires' disease can be severe and include respiratory failure, septic shock, and acute kidney failure. Indeed, mortality rates as high as 8.2–13.5% have been reported in infected individuals among the general populations of several European countries [24,25]. Studies indicate a dose dependent relationship between frequency of Legionnaires disease and passive smoking in children [26] as well as in adult smokers [27].

### Tuberculosis

More than 30% of world's population may be infected with the bacterial agent of tuberculosis, *Mycobacterium tuberculosis*. Tuberculosis represents the leading cause of death from infectious disease worldwide and the leading cause of death among HIV-positive individuals [28]. Smoking increases susceptibility to bacillary tuberculosis in a dose-dependent manner [29,30], adversely affects clinical manifestations [31], and accounts for 12% of all tuberculosis deaths [28]. The disease progresses more rapidly in smokers than in non-smokers, while smoking is also associated with increased relapse and mortality [32]. Quitting smoking and prevention of exposure to secondhand smoke are both important measures in the control of tuberculosis [33]. Indeed, the W.H.O. has estimated that, in China, heavy smoking (> 20 cigarettes per day) leads to a doubling of the death rate from tuberculosis [28].

### Bacterial meningitis

Carriage of *H. influenzae*, pneumococcus, and meningococcus has been shown to be more common in both active and second-hand smokers than in nonsmokers [34-38]. Indeed, the relationship may be dose-dependent and, in a study of military recruits, smoke exposure gave an attributable risk for meningococcal carriage of 33% [38]. In addition to increased carriage, meningitis cases have a two-to fourfold higher risk of exposure to cigarette smoke than controls [34]. Furthermore, second-hand smoke exposure predisposes to nasopharyngeal colonization with specific *Staphylococcus aureus *variants that may have altered capacity to compete with pneumococci subtypes [39]. Passive exposure to tobacco smoke has also been associated with *Hemophilus influenzae *and pneumococcus meningitis in Australian children [4].

### Vaginosis and sexually transmitted infections

Bacterial vaginosis, although often asymptomatic, can cause considerable discomfort and is associated with the development of more serious infections, such as septicemia and increased risk of poor pregnancy outcome [40-42]. Tobacco smoking has been significantly correlated with bacterial vaginosis, typically being in the region of twice as common in smokers as non-smokers, with a greater prevalence noted in young women [43-45]. Tobacco use has also been independently associated with a higher prevalence of specific sexually-transmitted bacterial infections – chlamydia and gonorrhoea [46].

### Helicobacter pylori infection

There are several factors that may contribute to an increased susceptibility to gastric and duodenal ulcers in smokers compared to non-smokers. Smoking decreases antacid (bicarbonate) secretion from the pancreas, while stimulating gastric acid secretion [47,48]. Furthermore, the great majority of gastric and duodenal ulcers are associated with *H. pylori *infection and smokers are more susceptible than non-smokers to gastrointestinal infection with this bacterial species [49-51]. Additionally, smoking has been significantly associated with the failure of anti-*H. pylori *therapy [52,53].

### Periodontitis

Patients who smoke exhibit not only increased susceptibility to periodontitis but are also more likely than non-smokers to display severe disease and to be refractory to treatment [54]. Despite some conflicting data that smoking may not influence the sub-gingival micoflora [55-58], the balance of recent data strongly suggests that tobacco-induced susceptibility to periodontitis is associated with shifts in the microbial composition of complex periodontal plaque communities smokers [51,59-66], as we have recently reviewed [54]. For example, Zambon et al showed a higher prevalence of *Aggregatibacter actinomycetemcomitans*, *Tannerella forsythia *and *Porphyromonas gingivalis *in smokers [59]. Umeda et al [63] reported an increased risk for harbouring *Treponema denticola *in smokers. Haffajee and Socransky [67] showed an increased prevalence of eight species, including *P. gingivalis*, in current smokers, while Eggert et al [62] have shown a higher prevalence and proportion of *T. forsythia*, *Campylobacter rectus*, *P. gingivalis *and *Peptostreptococcus micros *in plaque samples from smokers.

### Inflammatory bowel diseases

Of all the factors associated with inflammatory bowel diseases, the most solid evidence is for smoking and enteric bacterial flora – and these two risk factors may well be related [68]. Smoking increases the risk of Crohn's disease and worsens its clinical course, but has a protective effect in ulcerative colitis [68], as we and others have recently reviewed [69-71]. Furthermore, in ulcerative colitis patients, ex-smokers are more likely than current smokers to exhibit increased disease progression, more frequent hospitalization, and are twice as likely as current smokers to require colectomy [72]. However, in Crohn's disease patients, smokers exhibit higher disease recurrence rates, require more frequent surgical interventions, and have a greater need for immunosuppressive agents compared to non-smokers [72].

### Otitis media

Otitis media is an infection of the middle ear, commonly caused by *Moraxella catarrhalis*, *S. pneumoniae *and *H. influenzae*, that is rarely associated with severe complications, such as hearing loss and meningitis. The posterior nasopharynx flora of parents who smoke, compared to the flora of non-smoking parents, contains more potential pathogens similar to those recovered from otitis media-prone children [73]. Additionally, children exposed to secondhand smoke are more susceptible to otitis media [1]. Indeed, secondhand smoke has been estimated to be responsible for 0.7 to 1.6 million physician visits for middle ear infections annually in the USA alone [74].

### Post-Surgical and nosocomial infections

As many as 5% of patients undergoing surgery develop surgical site infections, which leads to significant morbidity and may sometimes be fatal [75]. Smoking is known to be an important patient-related risk factor for surgical site infections [75,76]. Smoking also increases the risk of nosocomial non-wound infections, bacteremia and urinary tract infections with multiple bacterial species and worsens bacteremic prognosis [77-80].

### In utero and neonatal second-hand smoke exposure, neonatal infection and sudden infant death syndrome (SIDS)

12% of US women smoke during pregnancy [81] and, conservatively, 50% of US children are exposed to secondhand smoke [82,83]. Maternal smoking has been associated with significantly higher rates of neonatal infection than found in the children of non-smoking mothers [82,84,85]. Furthermore, the odds ratios for SIDS in secondhand smoke-exposed neonates have risen considerably since the broad acceptance of "back-to-sleep" positioning [82]. It has recently been postulated that these two facts may be related and that SIDS could be caused by the interactions between components of cigarette smoke and infectious bacterial loads that would normally be sub-lethal in neonates that have not been exposed to tobacco smoke [82,86-88].

## Mechanisms of increased susceptibility to bacterial infections in smokers

There is a large and productive tobacco research literature that focuses on epidemiology, behavior, addiction and quitting. However, despite the wealth of epidemiological evidence of the profound ill-effects of smoking on human health, studies that set out to understand the mechanisms of *how *cigarette smoke induces disease are limited, as we have recently commented [89]. It is not the intent of this review to provide comprehensive data on why tobacco use leads to increased rates of infectious disease in smokers. Rather, we will introduce some of the key potential mechanisms underlying this increased susceptibility and provide the reader with links to more exhaustive literature reviews.

Cigarette smoking can, theoretically, increase the risk of infection by pathogenic or opportunistic bacteria by three general mechanisms:

• Tobacco-induced physiological and structural changes in humans.

• Tobacco-induced increase in bacterial virulence.

• Tobacco-induced dysregulation of immune function.

Such mechanisms are not mutually exclusive and all three may occur simultaneously. For example, tobacco smoke exposure may play a direct role in bacterial colonization of the respiratory tract by hindering mucociliary clearance of bacteria [19,90]; while inducing bacterial components that aid in the binding of microbes to respiratory epithelial cells ([7] and our own, unpublished data); and concurrently decreasing the ability of respiratory phagocytic cells to detect and destroy pathogenic microbes [91]. Tobacco-induced physiological and structural changes in humans have focused primarily on the vasculature and respiratory tract. The vasoactive effects of cigarette smoke and nicotine appear to vary in different vascular beds. For example, smoking induces vasoconstriction in peripheral arteries [92] but vasodilation in cerebral blood vessels [93]. In periodontal tissues smoking does not seem to exert an acute vasoactive (constriction or dilation) influence on the microvasculature [94]. Rather, smoking results in a suppression of periodontal angiogenesis [95] in a manner that is rapidly reversible on smoking cessation [96]. Thus, the negative influence of smoking on mucociliary function most likely contributes to increased risk of bacterial infection by reducing the ability of the respiratory tract to clear pathogens, while the vasoconstrictive or anti-angiogenic influence of tobacco may contribute to increased susceptibility to bacterial infection by decreasing the effectiveness of inflammatory responses to pathogenic bacteria.

Several research groups have examined the interactions between infectious agents and cigarette smoke components. For example, Sayers et al. have demonstrated the potentiating influence of low levels of nicotine on staphylococcal and enterobacter toxins in studies addressing why passive exposure to cigarette smoke is a risk factor in sudden infant death syndrome [86,87]. The same group has also shown that both nicotine and cotinine exhibit lethal synergy with toxins produced by several periodontal pathogens (*Prevotella*, *Porphyromonas *and *Fusobacterium *species) in the chick embryo toxicity model. Wiedeman et al. have suggested that tobacco smoke exposure may represent a risk for establishment of a chronic reservoir of *C. pneumoniae *infection within respiratory epithelium [97,98]. Other groups have shown that exposure to cigarette smoke affects the growth of bacteria which may facilitate populational shifts in the microbial communities that colonize some human tissues. Zonuz et al reported that the growth of *Streptococcus mutans *and *S. sanguis*, two common oral bacteria, was stimulated by cigarette smoke [99]. In contrast, Ertel et al. showed that cigarette smoke inhibited the growth of Gram positive organisms, e.g., *S. pneumoniae *and *S. aureus*, but had little effect on Gram negative enteric bacteria such as *Klebsiella, Enterobacter *and *Pseudomonas *[100]. Consistent with this observation, they report that smokers have a propensity to develop heavy Gram negative colonization of the oral cavity relative to non-smokers. Interestingly, women who smoke are at higher risk of contracting bacterial vaginosis, as discussed above. Pavlova and Tao showed that the trace amounts of benzo [a]pyrene diol epoxide that are present in the vaginal secretions of women who smoke promotes the induction of bacteriophages in resident lactobacilli [101]. This may lead to a reduction in lactobacilli populations and facilitate overgrowth of anaerobes that are associated with vaginosis. In general, however, mechanistic studies to examine the direct influence of tobacco smoke on bacterial physiology and their pathogenic potential are lacking in the literature.

### Dysregulation of innate immune function

Several innate cell receptor-tobacco agonist couples have been identified, suggesting that tobacco smoke is capable of effecting neutrophil and monocyte function both directly and indirectly [54,102]. Indeed, multiple effector functions of professional phagocytic and antigen presenting innate cells (neutrophils, monocytes, macrophages and dendritic cells) are compromised by tobacco smoke. For example, in neutrophils, tobacco smoke and/or nicotine have been shown to reduce key anti-microbial activities including phagocytosis (the engulfment and uptake of bacteria) [54,103,104]; the generation of a respiratory burst (the combined oxygen-dependent processes by which neutrophils kill phagocytosed bacterial cells) [102,105-107]; and, ultimately, the ability to kill specific bacterial species [102,106]. Compared to unexposed monocytic cells, tobacco-exposure suppresses general responsiveness to bacteria and lipopolysaccharide (LPS) [108], reflected in a down-regulation of surface pathogen recognition receptors (TLR-2 and MARCO) [109,110]; with reduced phagocytic, reactive-oxygen species generating, and bacterial killing capacities also reported [105,106,111]. Dendritic cells, whose primary function is to process antigen and present them to adaptive immune cells thus bridging innate and adapative immune responses, are also negatively influenced by tobacco smoke and smoke constituents. For example, nicotine exposure suppresses the maturation dendritic cells that, subsequently, exhibit a reduced expression of antigen presenting and costimulatory molecules (MHC Class II, CD80 and CD86), reduced capacity for antigen uptake and reduced production of T cell stimulating cytokines in response to the archetypal Gram-negative pro-inflammatory stimulus, LPS [112,113].

### Dysregulation of adaptive immune function

The potential effects of smoking on lymphocyte function are not well understood. However, while IgE levels are increased in smokers compared to non-smokers, concentrations of anti-bacterial IgG levels are reduced [114-118]. This may represent a key underlying mechanism of increased susceptibility to bacterial infection in smokers. Furthermore, in order to mount a successful humoral immune response, B cells require T helper cell-derived cytokines to proliferate and differentiate into plasma cells and to promote immunoglobulin class switching. However, it has been shown by several groups that tobacco smoke reduces T cell proliferative responses to mitogen/antigen [119-121], with similar tobacco-induced reductions in B cell proliferative responses also reported [122]. For further details of the influence of tobacco smoke on the immune system, we point the reader to several extensive reviews [54,69,71,123-129].

## Summary

The available evidence supporting tobacco smoke as a risk factor for multiple and varied bacterial infections is convincing. A summary of bacterial infections associated with tobacco smoking [2,4,5,22,28,38,44,51,130-138] is presented in Table 1. Additional references are provided for more complete information (nasopharyngeal and respiratory pathogens [1-3,5,6,9,13,14]; Group A streptococcus [15]; Legionnaires disease [5,23]; cystic fibrosis [17,18]; pneumonia [2,20,21]; tuberculosis [30-33]; meningococcal carriage [4,34-37,39,139]; bacterial vaginosis [43,45,46]; periductal mastitis [140,141]; *Helicobacter pylori *[49,50,52,53]; periodontitis [54,123,142-151]; ulcerative colitis [71,152,153]; Crohn's disease [152,154,155]; otitis media [1,73,156]; and surgical infections [75,76,80]).

**Table 1:** Bacterial infections associated with tobacco smoking.

**Infection**	OR (95% CI)*
Nasopharyngeal and respiratory pathogens (such as *S. pneumonia*, *N. meningitidis*, *H. influenzae*, *L. pneumophila*)	2.5 (1.1–6.0) [4]

Group A streptococcus sore throat	-

Legionnaires disease	3.6 (2.1–5.8) [5]

Cystic fibrosis	*Increased severity on smoke exposure *[134,138]

Pneumonia	2.6 (1.9–3.5)[22,137]

Tuberculosis	1.8 *Light *[29]
	3.2 *Moderate *[29]
	3.7 *Heavy *[29]
	
	4.1 (2.4 to 7.3) *Active *[2]
	2.5 (1.2 to 5.1) *Secondhand *[2]

Meningococcal carriage	2.2 (1.0–4.8) *Light *[38]
	7.2 (2.3–22.9 *Heavy *[38]

Bacterial vaginosis	2.7 [44]

Periductal mastitis	6.2 (2.9–13.4) [131]

*Helicobacter pylori*	1.9 (1.4–2.5) [51]

Periodontitis	3.3 (2.3–4.5) *Light *[132]
	7.3 (5.1–10.3) *Heavy *[132]

Ulcerative colitis	0.6 (0.4–0.8) [70]

Crohn's disease	3.6 (2.5 – 5.0) *Active *[130]
	2.0 (1.3 – 3.3) *Secondhand *[135]

Otitis media	4.2 (1.5–11.9) *Secondhand *[133]

Surgical infections	1.2 (1.1, 1.3) [136]

* The specific OR (95% CI) presented is selected from a single reference/study. Additional references are provided in the text for more complete information.

Nevertheless, there are several limitations to many individual studies. For example, some studies have small subject numbers. Others have found associations between tobacco smoke exposure and infection during in studies designed to answer different specific questions. Importantly, many additional factors are independently associated with bacterial infection, including age, socioeconomic status, healthcare provision, alcohol, low physical activity, certain sexual behaviors, and not all studies reporting on the associations between smoking and infection have addressed all confounders.

It is also important to note that in the existing literature on mechanisms of tobacco-induced disease, there is an overbearing focus on nicotine. While this approach is valid, it must be remembered that tobacco smoke contains more than 4000 chemicals and there is an urgent need for studies that examine other components of cigarette smoke as well as whole mainstream and side stream smoke preparations. To this end, there remains a pressing need for consensus on the standardization of *in vivo *and *in vitro *modeling systems to permit the generation of a more robust and reproducible evidence base.

We hope that this review will serve to generate a wider understanding of the increased susceptibility to infectious diseases in smoke-exposed individuals; provide further impetus to efforts aimed at reducing smoke exposure; and act as a stimulus for further epidemiological and, particularly, mechanistic research into this important area.

## Competing interests

The authors declare that they have no competing interests.

## Authors' contributions

This review article was invited by the editor of *Tobacco Induced Diseases *(J. Elliott Scott). JB, DRD and DAS all contributed to the analysis of the literature and participated in the design and coordination of the review. All authors read and approved the final manuscript.
